# The Soluble Secretome and Extracellular Vesicles of Synovial Fluid Cells Closely Resemble Synovial Membrane Fibroblasts over Articular Chondrocytes in Donor-Matched Isolates from Osteoarthritis Patients

**DOI:** 10.3390/jcm15218403

**Published:** 2026-10-28

**Authors:** Enrico Ragni, Paola De Luca, Michela Maria Taiana, Elena Della Morte, Federico Valli, Laura de Girolamo

**Affiliations:** 1 Laboratorio di Biotecnologie Applicate all’Ortopedia, IRCCS Ospedale Galeazzi—Sant’Ambrogio, Via Cristina Belgioioso 173, 20157 Milano, Italy; deluca.paola@grupposandonato.it (P.D.L.); michelamaria.taiana@grupposandonato.it (M.M.T.); laura.degirolamo@grupposandonato.it (L.d.G.); 2 Laboratory of Biotechnological Applications, IRCCS Ospedale Galeazzi—Sant’Ambrogio, Via Cristina Belgioioso 173, 20157 Milano, Italy; elena.dellamorte@grupposandonato.it; 3 Department of Chirurgia Articolare Sostitutiva e Chirurgia Ortopedica, IRCCS Ospedale Galeazzi—Sant’Ambrogio, Via Cristina Belgioioso 173, 20157 Milano, Italy; vallifederico@libero.it

**Keywords:** osteoarthritis, chondrocytes, fibroblast-like synoviocytes, synovial fluid cells, inflammation, extracellular matrix, extracellular vesicles, miRNA, secretome

## Abstract

**Background**: Osteoarthritis (OA) is a whole-joint pathological condition driven by an intricate signaling network among immune cells, articular chondrocytes (ACs), fibroblast-like synoviocytes (FLSs) and non-immune synovial fluid cells (SFCs). While AC and FLS secretomes are well-characterized, the phenotypic identity of SFCs and their signaling cross-talk with structural joint cells remain poorly defined. This study aimed to comparatively characterize the secretome and extracellular vesicles (EVs) profile of donor-matched human ACs, FLSs and SFCs to identify shared and cell-specific signaling features within the osteoarthritic joint. **Methods**: Primary donor-matched ACs, FLSs and SFCs were isolated from five human osteoarthritic joints. EVs and soluble secretomes were isolated and analyzed by EV surface marker immunophenotyping, EV-microRNAs (miRNAs) profiling and soluble factor analysis. Data were evaluated using principal component analysis, hierarchical clustering, correlation matrices and bioinformatic analyses. **Results**: All three cell types shared a baseline OA secretome fingerprint dominated by inflammatory and catabolic factors. However, AC secretomes segregated distinctly. FLSs and SFCs displayed high phenotypic correlation across EV surface markers (r = 0.95), EV-miRNA signatures (r = 0.94) and soluble factor profiles (r = 0.94). Bioinformatic analyses showed that upregulated EV-miRNAs shared between FLSs and SFCs were specifically involved with pathways related to immune cell activation, cytokine signaling and apoptosis. In contrast, ACs demonstrated a distinct profile marked by the significant upregulation of soluble catabolic and pro-inflammatory mediators. **Conclusions**: Non-immune SFCs closely resemble FLSs in their secretome and EV profiles, supporting a similar phenotype and a potential role in EV-mediated immune and inflammatory signaling. In contrast, ACs display a distinct profile characterized by soluble catabolic and pro-inflammatory mediators. These findings highlight compartment-specific signaling patterns within the osteoarthritic joint and may inform future cell-free therapeutic strategies.

## 1. Introduction

Osteoarthritis (OA) is a whole-joint pathological condition characterized by progressive cartilage loss, subchondral bone remodeling, synovial tissue inflammation and disrupted intra-articular homeostasis [1]. Far from being a mere degeneration of articular cartilage, OA is driven by an intricate and altered signaling network among the structural and fluid compartments of the joint capsule [2]. Within this enclosed biochemical ecosystem where inflammatory and immune cells play a well-studied key role in sustaining joint pathology progression [3], articular chondrocytes (ACs) [4], synovial membrane fibroblasts (fibroblast-like synoviocytes (FLSs)) [5] and non-immune synovial fluid cells (SFCs) [3] continuously interact.

Distinct from transient immune cells recruited to the synovial fluid during inflammation, SFCs represent a non-immune connective tissue-type population with a mesenchymal stromal cell-like phenotype that can adhere to plastic surfaces in vitro, display a spindle-shaped fibroblast-like appearance and phenotype and show the reactivity of tissue synoviocytes [6]. These cells are also hypothesized to play an active role in local joint regulation and homeostasis [7]. Whether these floating stromal cells originate from the adjacent synovium [8], represent an endogenous cell pool attempting cartilage repair [9] or express a distinct phenotypic identity shaped by the osteoarthritic fluid environment [3] remains an open question. Thus, establishing how SFCs relate to their structural counterparts may be essential for understanding joint homeostasis and tissue-cell interactions in joint pathology. In this context, intercellular communication among these three stromal populations is heavily mediated by paracrine mechanisms, including soluble factors [10] such as cytokines, chemokines and growth factors and extracellular vesicles (EVs) [11], which serve as essential signaling vectors carrying surface antigens [12] and regulatory non-coding RNAs [13], particularly microRNAs (miRNAs) [14]. Collectively, soluble factors and EVs define the secretome [15], which is highly cell- or tissue-of-origin-specific [16].

In the context of joint disease, the EV cargo and secretome of AC [17,18] and FLS [19,20] have been mapped and compared to study the cartilage-synovia crosstalk [21,22]. In contrast, SFCs have largely been studied as stand-alone players, and their secretome has not been systematically compared with those of ACs and FLSs within the same osteoarthritic joint. Consequently, the similarities and differences between all these cell types, and in particular the potential contribution of SFCs to intra-articular cellular crosstalk, remain poorly understood.

To address this knowledge gap, the present study comprehensively profiled the secretomes of donor-matched SFCs, FLSs and ACs isolated from human osteoarthritic joints. By cross-analyzing the EV surface markers, EV-miRNA expression signatures and secreted soluble factor landscapes, we aimed to elucidate shared and cell-specific communication features among these three joint cell populations, with a focus on determining whether the SFC secretome more closely resembles the FLS or AC profile. Shedding light on these tissue-cell relationships may improve our understanding of intra-articular signaling in OA and provide a biological basis for the selection of optimal cell or cell-free candidates for musculoskeletal biotherapies.

## 2. Materials and Methods

### 2.1. Articular Chondrocytes, Synovial Membrane and Synovial Fluid Cell Isolation and Expansion

Human biological specimens were collected as discarded surgical tissue from five osteoarthritic donors (3 females, 2 males; overall mean age: 68 ± 6 years) presenting with end-stage disease (Kellgren–Lawrence grade III–IV) and undergoing elective total knee arthroplasty.

Articular chondrocytes (ACs) were isolated from morphologically undamaged areas of articular cartilage after enzymatic digestion with 0.15% (*w*/*v*) Type II Collagenase (Worthington Biochemical Corporation, Lakewood, NJ, USA) for 22 h at 37 °C under continuous orbital shaking. Liberated ACs were subsequently propagated in high-glucose Dulbecco’s Modified Eagle Medium containing 10% (*v*/*v*) fetal bovine serum (FBS), 1% (*v*/*v*) L-glutamine (200 mM), 1% (*v*/*v*) penicillin/streptomycin solution (10,000 U/mL penicillin, 10 mg/mL streptomycin) and 1% (*v*/*v*) amphotericin B (250 µg/mL) (all culture reagents sourced from Thermo Fisher Scientific, Waltham, MA, USA). Cell cultures were maintained in a humidified incubator (37 °C, 5% CO_2_) until attaining ~80% confluence prior to subculturing or cryopreservation in liquid nitrogen.

Fibroblast-like synoviocytes (FLSs) were isolated from synovial tissue fragments, which were minced and enzymatically digested with 0.25% (*w*/*v*) Type I Collagenase (Worthington Biochemical Corporation) for 3 h at 37 °C. The resulting single-cell suspension was passed through a 100 µm nylon mesh strainer to remove undigested tissue debris. Isolated FLSs were expanded using the exact formulation and culture conditions detailed above for ACs. At the time of experimental procedures, cells at 80% confluence were stained with Alcian Blue (Bio Optica Milano S.p.A., Milano, Italy) following the manufacturer’s instructions. After dehydratation, staining was documented.

Synovial fluid cells (SFCs) were obtained from intra-articular synovial fluid aspirates. The fluids were diluted in complete high-glucose DMEM and seeded into tissue-culture-treated plasticware to selectively harvest the plastic-adherent SFC fraction. Non-adherent cells were removed, and adherent stromal colonies were expanded following the same media and incubation protocol applied to ACs and FLSs.

All three cellular lineages were standardized and utilized at passage 5 for downstream experimental assays after confirmation of cell identity by flow cytometry. Briefly, 50,000 cells were left unstained or stained at 4 °C in the dark with the following antibodies for ACs (anti-CD90-FITC, anti-CD73-PE, anti-CD45-PE-Vio770, all from Miltenyi, Bergish Gladbach, Germany, and anti-CD44-APC, from BioLegend, San Diego, CA, USA), or FLSs and SFCs (anti-CD90-FITC, anti-CD73-PE and anti-CD45-PE-Vio770, and anti-CD14-PC5 from BioLegend). After one wash in FACS buffer, cells were analyzed with a CytoFlex flow cytometer (Beckman Coulter, Brea, CA, USA), acquiring at least 20,000 events.

### 2.2. Secretome Production

All cell types were seeded at an initial density of 10,000 cells/cm^2^ in complete medium. After 3 days, cell monolayers were thoroughly rinsed three times with PBS to remove residual medium, after which serum-free DMEM was introduced. Cultures were incubated for an additional 48 h to generate conditioned media (secretome). To clarify the secretome of intact cells and cellular remnants, collected supernatants were subjected to serial centrifugation steps: 376× *g* for 1 cycle, 1000× *g* for 1 cycle, 2000× *g* for 1 cycle, and two consecutive rounds at 4000× *g*. Cleared conditioned media were immediately stored at −80 °C for future experimental evaluations. Cell number and viability were assessed after secretome removal with a NucleoCounter NC-3000 (ChemoMeTec, Allerød, Denmark). A 95% viability level was used as a benchmark to proceed with further secretome testing.

### 2.3. Extracellular Vesicles Analysis

Secretome samples were diluted 1-fold in PBS to a total volume of 10 mL and subjected to ultracentrifugation at 100,000× *g* for 9 h at 4 °C to pellet extracellular vesicles (EVs). Centrifugation was carried out using an Optima L-90K Ultracentrifuge (Beckman Coulter) fitted with a Type 70.1 Ti fixed-angle titanium rotor (Beckman Coulter). EVs were studied using three approaches:

(i) Flow cytometric surface marker profiling: Surface expression markers on EVs were analyzed using the MACSPlex EV Kit IO human (Miltenyi Biotec) following the protocol provided by the supplier. Multiplexed marker detection was executed on a CytoFLEX flow cytometer (Beckman Coulter). Data normalization was performed on raw median fluorescence intensity values. Background subtraction was initially executed by deducting the median signal intensity of each bead population in the buffer-only control sample from the corresponding bead intensities of the experimental samples. For each individual sample, the signal intensities corresponding to the internal pan-EV markers (CD9, CD63 and CD81) were extracted, and their arithmetic median was calculated to establish a sample-specific normalization factor. The relative expression of each surface marker was subsequently determined by calculating the ratio of the normalized signal intensities between the target experimental samples.
(ii) Nanoparticle tracking analysis (NTA): EV particle yields and size distribution measurements were evaluated using a NanoSight NS300 instrument (NanoSight Ltd., Amesbury, UK). EV samples were diluted in PBS to achieve an optimal particle density range of 20 to 120 particles per frame. Five independent 60 s video recordings were captured per sample, and data were processed using NTA software version 3.4 (NanoSight Ltd.).
(iii) Transmission electron microscopy (TEM): Morphological assessment was performed by placing a 5 µL droplet of EV suspension onto formvar/carbon-coated electron microscopy grids for 10 min at room temperature. Liquid excess was carefully removed using filter paper. Negative staining was subsequently applied using a 2% (*w*/*v*) uranyl acetate aqueous solution for 10 min, after which surplus stain was blotted away. Following ambient air-drying, grids were visualized under a TALOS L120C transmission electron microscope (Thermo Fisher Scientific) operating at an accelerating voltage of 120 kV. Dimensional data were exported as mean diameter ± standard deviation (SD).
(iv) Purity: EV fractions were analyzed with a NanoDrop ND-1000 spectrophotometer (Thermo Fisher Scientific) with the Protein A280 protocol. The concentration was related with NTA data to obtain the number of EVs/µg protein value.
(v) Western blot analysis: 7.5 µg of EVs and cell lysate in RIPA buffer + protease inhibitors quantified with BCA (Cyanagen, Bologna, Italy) were loaded onto a 12% SDS–polyacrylamide gel and separated by electrophoresis. Western blotting was performed according to standard procedures. The membranes were incubated overnight at 4 °C with the following primary Abs: mouse anti-Flotillin-1 (610820; BD Transduction Laboratories, Franklin Lakes, NJ, USA; 1:500), rabbit anti-TSG101 (HPA006161; Merck, Darmstadt, Germany; 1:1000), rabbit anti-Calnexin (TA336279; OriGene Technologies, Rockville, MD, USA; 1:1000) and rabbit anti-CD9 (ExoAb Antibody Kit; System Biosciences, Palo Alto, CA, USA; 1:1000). Signals were detected using appropriate HRP-conjugated secondary Abs (Thermo Fisher Scientific; 1:2000 or ExoAb Rabbit Secondary Antibody System Biosciences, Palo Alto, CA, USA; 1:10,000) and visualized with Westar Supernova enhanced chemiluminescence substrate (Cyanagen). Chemiluminescent signals were acquired using a ChemiDoc imaging system (Bio-Rad Laboratories, Hercules, CA, USA).

### 2.4. ELISA Characterization of Secretomes

Secretome samples were analyzed using the multiplexed enzyme-linked immunosorbent assay (ELISA) Quantibody Human Cytokine Array 4000 Kit (RayBiotech, Peachtree Corners, GA, USA), which assesses the abundance of 200 soluble factors, in accordance with the manufacturer’s instructions. One-fold diluted secretomes in binding buffer were processed, and fluorescence signal intensities were quantified. To ensure analytical rigor, downstream comparative analyses included only analytes that exceeded the assay’s lower limit of detection across all evaluated samples or exhibited cell type-specific detection (i.e., present in all samples of one or more cell types and below the lower limit of detection in all samples of the remaining group(s)). After applying the appropriate dilution adjustments, final protein concentrations were normalized to total cell numbers and expressed as pg per 10^6^ cells.

### 2.5. EV-miRNA Detection

Total RNA extraction from pelleted EVs, reverse transcription for cDNA synthesis and real-time quantitative PCR (qRT-PCR) assays were conducted following previously established protocols [23]. Technical efficiency across processing steps was monitored by incorporating exogenous *Arabidopsis thaliana* miR-159 (ath-miR-159) as a spike-in control to equalize panels A and B of the OpenArray system (Life Technologies, Foster City, CA, USA), which determines miRNA expression in 384-well OpenArray plates for a total of 768 calls, according to the manufacturer’s protocol. Each single miRNA was considered present when amplification resulted in all three samples. Data normalization was executed using the global mean method [24], and only microRNAs displaying cycle threshold (C_RT_) values ≤ 35 across all evaluated samples or exhibiting cell type-specific detection (i.e., present in all samples of one or more cell types and below the lower limit of detection in all samples of the remaining group(s)) were retained for quantitative evaluation. Relative fold-change values were calculated using the 2^(-Delta C_RT_) method, accounting for the inverse relationship between C_RT_ values and expression levels, where ratios ≥ 2.0 indicate upregulation/enrichment and ratios ≤ 0.5 indicate downregulation.

### 2.6. Clustering Analyses

Multivariate data exploration, including principal component analysis (PCA) and hierarchical clustering analysis, was performed using the ClustVis v.beta web tool platform (v.beta; https://biit.cs.ut.ee/clustvis/; accessed on 30th July 2026) [25]. Prior to analysis, quantitative data were used untransformed (C_RT_ for EV-miRNAs) or natural log-transformed [ln(x) for ELISA analytes or ln(x + 1) for EV immunophenotype data to account for MFI range discrepancies, as well as for the merged dataset to harmonize signal magnitudes across the three heterogeneous data blocks when values equal or close to 0 were present in at least one sample]. No row centering or row scaling parameters were applied to the datasets. Principal components were calculated using singular-value decomposition with imputation. For heatmap generation and unsupervised hierarchical clustering, both rows and columns were ordered using correlation distance metrics and average linkage criteria. Clustering trees for rows and columns were arranged according to the tightest cluster first ordering parameter.

### 2.7. Computational Analysis of EV-miRNAs

Analysis of miRNA (disease, pathway and gene ontology) was performed via the Over-Representation Analysis (ORA) method of the miRNA Enrichment Analysis and Annotation Tool (miEAA, v.2.1; https://ccb-compute2.cs.uni-saarland.de/mieaa; accessed on 6th August 2026). The following parameters were set: (i) *p*-value adjustment method: FDR (Benjamini-Hochberg) adjustment; (ii) significance level: 0.05; (iii) minimum required hits per sub-category: 2.

### 2.8. Protein–Protein Interaction Network Analysis

Protein–protein interaction networks for the ELISA-identified factors were constructed using the STRING database (v.12.0; http://www.string-db.org; accessed on 6th August 2026) [26]. Analysis parameters were set to *Homo sapiens*, utilizing a full STRING network that encompasses both physical and functional protein associations. Edge visualization was based on evidence type, with active interaction sources restricted to text mining. To ensure data reliability, a default high-confidence score threshold of 0.700 was applied (scores range from 0 to 1, indicating the statistical likelihood of true interactions). For biological processes analysis, a FDR ≤ 0.05 was set for significance.

### 2.9. Statistical Analyses

Statistical analyses were conducted using Prism software v.8 (GraphPad Software, La Jolla, CA, USA). Values after normalization (nm for EV size, fluorescence for EV markers, C_RT_ for EV-miRNAs and pg per 10^6^ cells for ELISA) were used. For all datasets, a Shapiro-Wilk test for normal distribution was performed (alpha = 0.05). If passed, a RM one-way ANOVA was conducted with Tukey correction for multiple comparisons. Otherwise, a Friedman test was run with Dunn correction for multiple comparisons. These post hoc corrections were applied for pairwise comparisons between cell groups per analyte, whereas no global correction across the entire panel of analytes was performed. For correlation analyses, a Shapiro–Wilk test for normal distribution was performed (alpha = 0.05). If passed, a Pearson correlation coefficient was calculated. If not passed, a nonparametric Spearman correlation was calculated. Data are reported as mean ± SD. Significance is reported as *p*-value ≤ 0.05 coupled with a ratio ≥ 2.0 or ≤0.5.

## 3. Results

### 3.1. AC, FLS and SFC Immunophenotype

ACs, FLSs and SFCs were positive for the stromal markers CD73 and CD90, and negative for the hematopoietic marker CD45 (Figure 1). ACs were also positive for the lineage marker CD44 and strongly positive for Alcian Blue staining (Supplementary Figure S1), suggesting active matrix deposition and the absence of major de-differentiation due to passaging. Furthermore, FLS and SFC samples lacked the expression of CD14, a lineage marker of monocytes that can be co-isolated by plastic adherence and detected in early passages, showing the purity of isolated cultures (Figure 1). Taken together, these profile characteristics confirm the non-immune, connective-tissue phenotype of FLSs and SFCs used in the study.

### 3.2. EV Characterization

Nanoparticle tracking analysis (NTA), used to evaluate the hydrodynamic size distribution and concentration of extracellular vesicles (EVs), showed that EVs derived from FLSs and SFCs exhibited significantly larger mean particle diameters compared to AC-derived EVs (195 ± 1 nm and 188 ± 7 nm vs. 167 ± 3 nm, respectively; Figure 2A,B). Transmission electron microscopy confirmed the characteristic nanoscale cup-shaped morphology and size range of the isolated vesicles across all cellular sources (Supplementary Figure S2). Although sample processing to obtain TEM images may alter EV dimensions, size analysis confirmed that AC-EVs were significantly smaller (AC-EVs: 71 ± 19 nm, FLS-EVs: 151 ± 33 nm, SFC-EVs: 140 ± 34 nm; AC-EVs vs. FLS-EVs *p*-value of 0.0001, AC-EVs vs. SFC-EVs *p*-value of 0.001, FLS-EVs vs. SFC-EVs *p*-value > 0.9999). The EV/μg protein ratio was 0.78 × 10^9^ ± 0.11 (AC), 0.85 × 10^9^ ± 0.13 (FLS) and 0.72 × 10^9^ ± 0.10 (SFC), the values falling within the range (10^8^ to 10^10^) reported for pure EV preparations from cell culture supernatants [27]. The particles consistently tested positive for the EV markers Flotillin-1, TSG101 and CD9, and negative for Calnexin, which is a marker for intracellular contamination (Figure 2C). Multiplexed immunophenotyping of EV surface markers highlighted a consistent stromal profile across the three cell sources. Among the 23 detected surface markers (Table S1), the stromal CD29, CD44, CD49e and CD105 antigens were uniformly expressed, whereas the hematopoietic marker CD45 was absent in all samples. PCA and unsupervised hierarchical clustering (Figure 2D,E) revealed distinct profile segregation for the three EV populations. Intra-source reproducibility was high within each EV type, with mean Spearman correlation coefficients of 0.95 ± 0.02 for AC-EVs, 0.98 ± 0.01 for FLS-EVs and 0.97 ± 0.02 for SFC-EVs. Inter-source comparisons further corroborated the clustering patterns, revealing a remarkably high correlation between FLS-EVs and SFC-EVs (0.95 ± 0.01), while correlations with AC-EVs were lower (0.84 ± 0.01 and 0.85 ± 0.02, respectively). Overall, 11 antigens were differentially expressed (Table 1). The most pronounced phenotypic differences were observed between FLS- and SFC-EVs and AC-EVs, with 9 and 8 differentially expressed markers, respectively. Conversely, only 4 surface markers differed between FLS-EVs and SFC-EVs. Notably, the epithelial CD326 marker was undetectable in all AC-EV samples, reflecting the pseudo-epithelial adhesion profile characteristic of the synovial lining and joint fluid-exposed cells, while the chondrocyte-associated marker CD146 was markedly downregulated in FLS-EVs and SFC-EVs compared to AC-EVs (45-fold decrease and 58-fold decrease, respectively). Taken together, these findings demonstrate that SFC-EVs phenotypically resemble FLS-EVs more closely than AC-EVs.

### 3.3. EV-miRNA Comparison

Across the EV samples, 167 associated miRNAs were detected (Table S2). Among the 10 most abundant miRNAs, all were common to both AC and SFC, with 9 also identified in the top 10 FLS-EVs (Table 2). Notably, several of these miRNAs are associated with OA and related biological processes (Table 2), including extracellular matrix disassembly (GO0022617), positive regulation of T cell and macrophage activation (GO0050870 and GO0043032), and induction of apoptosis (GO0006917), supporting the concept that OA involves multiple joint tissues.

In accordance with the shared message shuttled by the most abundant EV-miRNAs, PCA and hierarchical clustering on the entire dataset could not identify a clear dichotomy between FLS and SFC samples with respect to AC ones (Figure 3A,B). This was due to lower intra-sample reproducibility within each EV type, with mean Spearman correlation coefficients of 0.84 ± 0.07 for AC-EVs, 0.88 ± 0.05 for FLS-EVs and 0.85 ± 0.06 for SFC-EVs. Nevertheless, the inter-source comparisons again highlighted higher similarity for FLS-EVs and SFC-EVs (0.94 ± 0.01), whereas lower correlation values were observed when comparing FLS-EVs or SFC-EVs to AC-EVs (0.92 ± 0.03 and 0.89 ± 0.03, respectively). Accordingly, the analysis was then focused on significantly different miRNAs (Table 3). Notably, 42 miRNAs were differentially detected in at least one sample type. AC-EVs exhibited the most distinct expression profile, with 38 miRNAs differentially expressed compared to SFC and 23 compared to FLS. In contrast, only two miRNAs were differentially detected between SFC and FLS. Reinforcing the close similarity between FLS and SFC, even when differential expression did not reach statistical significance in the FLS vs. AC comparison, the direction of regulation remained identical. To explore the functional significance of miRNA regulation, over-representation pathway analysis was performed on the significantly upregulated miRNAs shared between FLS and SFC (Table S3). This analysis revealed enrichment in pathways involved with apoptosis, T and B cell activation, cytokine signaling (including IL-1, IL-3, IL-6 and IL-7) and the inflammatory response (Figure 3C). Notably, apoptosis pathways were associated with all 9 tested miRNAs, T cell activation with 8, cytokine signaling with 7 and inflammatory response with 5 (Figure 3D), pointing to a concerted biological message carried by abundant FLS- and SFC-derived EV-miRNAs. Conversely, over-representation analysis of the shared downregulated miRNAs yielded no significant pathway enrichment, indicating the absence of a distinct functional focus for AC EV-miRNAs.

### 3.4. Soluble Factor Comparison

Out of 200 analyzed factors, 27 secretome proteins, being consistently present across all samples or strictly group-specific, met our detection criteria and were selected for quantitative comparison (Table S4). Among the 10 most abundant factors, all were present in SFC secretomes, whereas 9 were identified in both FLS and AC top-10 lists (Table 4). Notably, the majority of factors are involved in OA-related processes, including inflammation (IGFBP6 and VCAM1), catabolism (IGFBP3 and 4), fibrosis (SERPINE1) and angiogenesis (VEGFA), while TIMP1 and 2 have a protective function on ECM. Thus, overall, as for EV-miRNAs, the most abundant proteins have a common preponderance for pathogenesis regardless of the joint tissue origin. This fingerprint was maintained when scoring the complete dataset (Table S5). Gene ontology mining showed that in the top 10 most significant and enriched biological processes, a network encompassed terms mainly related to inflammation (GO0006954, Inflammatory response) and cell chemotaxis (GO0050921, positive regulation of chemotaxis; GO0050920, regulation of chemotaxis), with a focus on leukocytes (GO0002688, regulation of leukocyte chemotaxis; GO0002690, Positive regulation of leukocyte chemotaxis) and monocytes (GO0002548, monocyte chemotaxis) (Figure 4A,B).

PCA and hierarchical clustering on the entire dataset were able to separate FLS and SFC samples from AC ones, which clustered more tightly (Figure 4C,D). This was confirmed by a high correlation within AC samples (Spearman 0.93 ± 0.04) with respect to FLS (0.91 ± 0.04) and SFC (0.88 ± 0.04) ones, which were more spread. Inter-source correlation analysis also confirmed the different AC fingerprint, with FLSs and SFCs being more similar (0.94 ± 0.0) than to AC (0.90 ± 0.03 and 0.90 ± 0.04, respectively). Accordingly, the analysis focused on significantly different players (Table 5). Notably, 6 soluble factors emerged, with 5 in the SFC vs. AC comparison, 3 in the FLS vs. AC comparison, and 2 shared by both FLSs and SFCs vs. ACs. Even when not concurrently significant, all factors showed a similar trend in both FLSs and SFCs with respect to ACs. Overall, modulated proteins show a preponderance of pro-OA signals in ACs, covering different aspects of pathologic cells such as catabolism (IGFBP4) and ECM degradation (PLAUR), inflammation (IL6R and TNFRSF1A) and immune cell recruitment (CCL26).

### 3.5. Overall Sample Comparison

EV immunophenotype, EV-miRNAs and soluble factors data were merged and analyzed. PCA clearly showed that AC samples sharply clustered apart (Figure 5A). FLS and SFC samples proved to be more similar and were in the same node in the hierarchical clustering, although a second-level separation emerged (Figure 5B). Taken together, these data emphasize the shared fingerprint of FLS and SFC with respect to AC samples.

## 4. Discussion

The cross-profiling of extracellular vesicle (EV) surface markers, EV-miRNA cargo and soluble secretome factors from donor-matched human osteoarthritic articular chondrocytes (ACs), fibroblast-like synoviocytes (FLSs) and synovial fluid cells (SFCs) revealed a shared core osteoarthritic fingerprint alongside distinct, lineage-specific signaling phenotypes.

Despite being isolated from separate intra-articular niches, all three cell populations shared key disease-associated signaling characteristics, reflecting the whole-joint nature of osteoarthritis (OA) [1]. The overarching secretome profile was dominated by drivers of cartilage catabolism, extracellular matrix disassembly and immune cell recruitment, mirroring crucial features in OA patients’ joints [28,29,30]. However, differential profiling revealed a distinct separation between tissue-resident chondrocytes and synovia-associated populations. ACs exhibited a highly specialized profile, marked by exclusive or elevated expression of specific markers such as CD146 and CD326 on EVs, as well as a distinct soluble factor release pattern. Conversely, FLSs and SFCs showed remarkable phenotypic and secretome similarity across all tested parameters, with SFCs consistently aligning with the synovial fibroblast lineage rather than forming an isolated cell entity. This is in agreement with previous data that fibroblast-like cells can be found in synovial fluid [31] and that these cells share functional similarity with FLSs from the synovial membrane [32]. Nevertheless, some differences between FLSs and SFCs were observed, possibly due to the direct influence of pathologic synovial fluid, as previously reported [33].

This phenotypic convergence between FLS and SFC vs. AC points to distinct yet complementary roles for these populations within the osteoarthritic microenvironment. FLSs and SFCs appear heavily dedicated to sustaining local inflammatory pathways and orchestrating interactions with immune cells. This is particularly evident in their EV-miRNA profiles: miRNAs upregulated in both FLSs and SFCs were uniquely enriched in functional pathways controlling T and B cell activation, cytokine responses (including IL-1, IL-3, IL-6 and IL-7) and apoptosis, all heavily modulated in OA [10,34,35,36,37,38]. Through these EV-encapsulated non-coding RNAs, FLSs and SFCs may contribute to surrounding cells and immune responses within the joint capsule [32,39,40]. This is in agreement with published data that showed that FLSs [20] and SFCs [32] are functionally altered in OA, resulting in an enhanced release of and response to pro-inflammatory factors in the joint environment to sustain the disease progression loop.

In contrast, ACs appear more directly involved in driving the local accumulation of soluble inflammatory mediators and catabolic factors within the articular matrix. Key soluble receptors and mediators, including IGFBP4, PLAUR, IL6R, TNFRSF1A and CCL26, showed upregulation in ACs compared to both FLSs and SFCs. While FLSs and SFCs deploy EV-miRNA signaling to regulate surrounding cells and immune responses, ACs actively release higher levels of soluble factors that sustain matrix degradation and amplify local pro-inflammatory signaling. These data are in agreement with the postulated critical roles of chondrocytes in initiating, sustaining and amplifying the inflammatory micro-environment of OA rather than being a passive end-target tissue [41]. In this frame, another crucial factor is the inflammation-driven release of cartilage fragments able to trigger the immune response that further enhances the production of catabolic factors and inflammation-related factors [42].

Ultimately, these findings highlight that OA pathogenesis relies on a tightly coordinated labor division among joint-dwelling cells. While overall secretome profiles share a baseline disease-associated fingerprint, the interplay between the EV-driven immuno-modulatory mechanisms of FLS/SFC and the soluble factor-driven catabolism of ACs illustrates how distinct structural compartments synergize to sustain joint-wide pathology.

The study presents some limitations. One limitation is that the cells were derived exclusively from end-stage osteoarthritic joints of patients who underwent total knee replacement, limiting our capacity to observe secretome kinetics across earlier, asymptomatic stages of disease progression. Second, cell expansion up to passage 5 under uniform culture conditions may have favored a phenotypic convergence between FLSs and SFCs. Nevertheless, the preservation of a distinct AC phenotype suggests that culture-induced convergence, if present, was restricted. Third, while functional enrichment analyses revealed clear regulatory networks for shared FLS- and SFC-derived EV-miRNAs, these pathways were identified based on bioinformatic prediction without direct in vitro functional validation on target cells, such as chondrocytes or immune cell subsets. Thus, all pathway-related discussion points remain candidate-generating and observational. Future studies utilizing functional approaches (such as EV uptake, reporter assays, or targeted gene knockdown) alongside microfluidic or co-culture model systems will be essential to validate these predicted biological effects and to establish the precise mechanistic pathways driven by these vesicular cargoes. In addition, to give a complete picture of the osteoarthritic joint, immune cells from both the synovium and the synovial fluid will have to be considered, because they are a crucial part of the feedback-loop mechanism initiating and sustaining OA pathology. Moreover, both miRNAs and soluble factors were tested in the frame of pre-defined panels to identify those molecules with a well-defined characterization and more powerful techniques, such as global miRNA sequencing or mass spectrometry-based proteomics, will have to be performed to identify less described or new miRNA or protein players. In this context, methodological limitations should also be noted. Although the donor-matched design increased statistical power, the small cohort size (n = 5) limits the robustness of normality distribution testing. Additionally, while strict pre-filtering and dual significance/magnitude thresholds were enforced, no global multi-analyte correction (such as FDR) was applied across the EV-miRNA and cytokine arrays. Consequently, potential false-positive findings cannot be entirely ruled out, and these high-throughput profiling data should be considered exploratory, paving the way for targeted validation studies in larger cohorts with untargeted analyses using next-generation technologies such as next-generation sequencing or proteomic approaches. Finally, in the integrative PCA and hierarchical clustering, datasets with different physical units were harmonized using log transformation without row scaling. While this preserved the relative signal magnitude of each analyte and avoided amplifying noise from low-abundance targets, the influence of residual variance differences across platforms should be taken into account when interpreting cluster separation.

## 5. Conclusions

In conclusion, this study shows that non-immune synovial fluid cells share a remarkably similar secretome and extracellular vesicle profile with fibroblast-like synoviocytes, supporting their close biological relationship and suggesting potential involvement in intercellular signaling and inflammatory crosstalk within the osteoarthritic joint. In contrast, articular chondrocytes display a distinct phenotypic identity, predominantly driven by the secretion of soluble catabolic and pro-inflammatory mediators. Together, these findings support distinct but coordinated compartment-specific signaling roles among joint cell populations in the osteoarthritic environment and contribute to a better understanding of the secretory profiles characterizing different joint-derived cell populations.

## Figures and Tables

**Figure 1:**
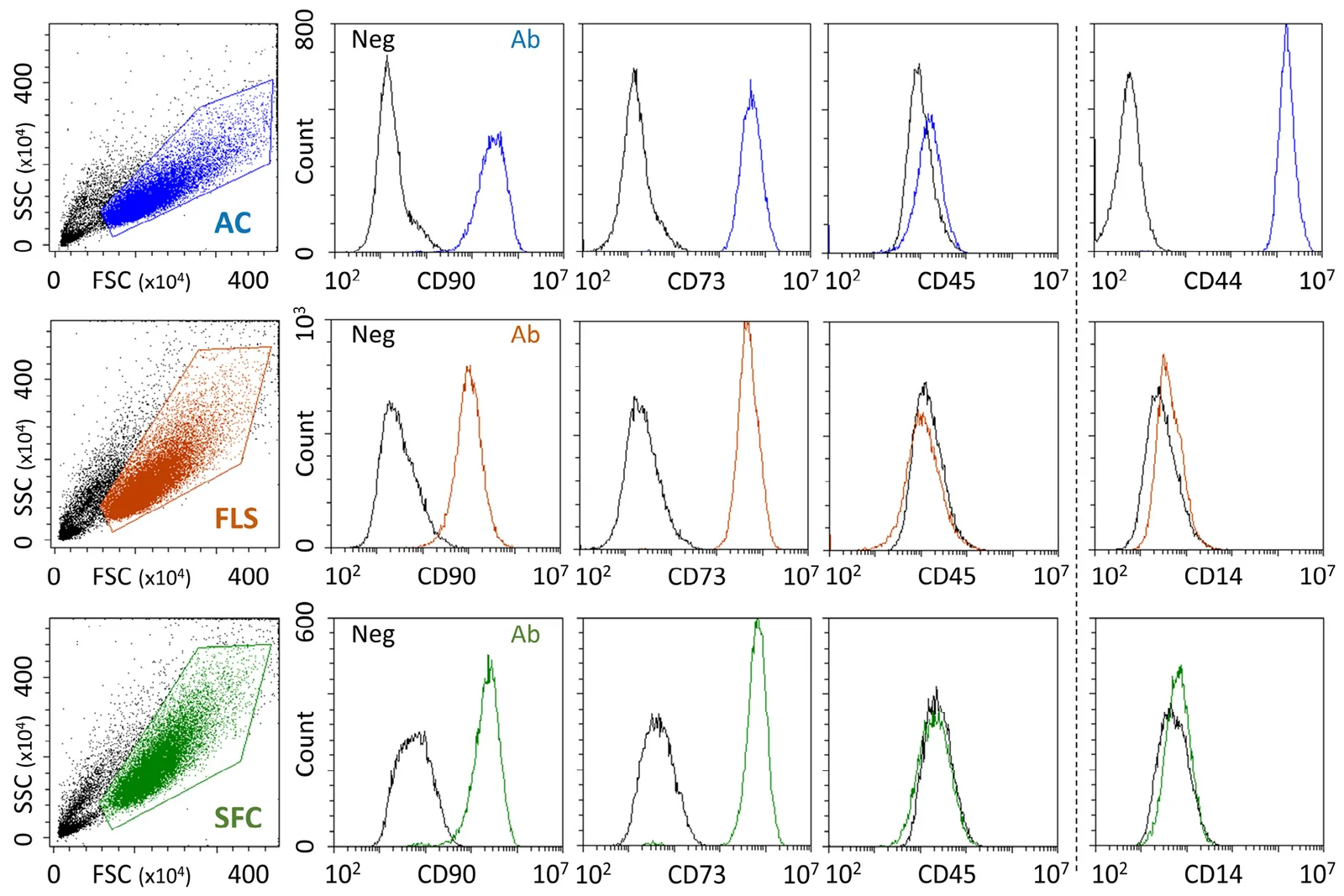
AC, FLS and SFC immunophenotype. Representative cytograms of AC, FLS and SFC samples stained for stromal CD90 and CD73 markers or the hematopoietic CD45 marker. ACs were also tested for the chondrocyte lineage CD44 marker, while FLSs and SFCs were tested for the monocyte lineage CD14 marker. In the overlay cytograms, unstained cells (Neg) and Ab-stained cells (Ab) are shown.

**Figure 2:**
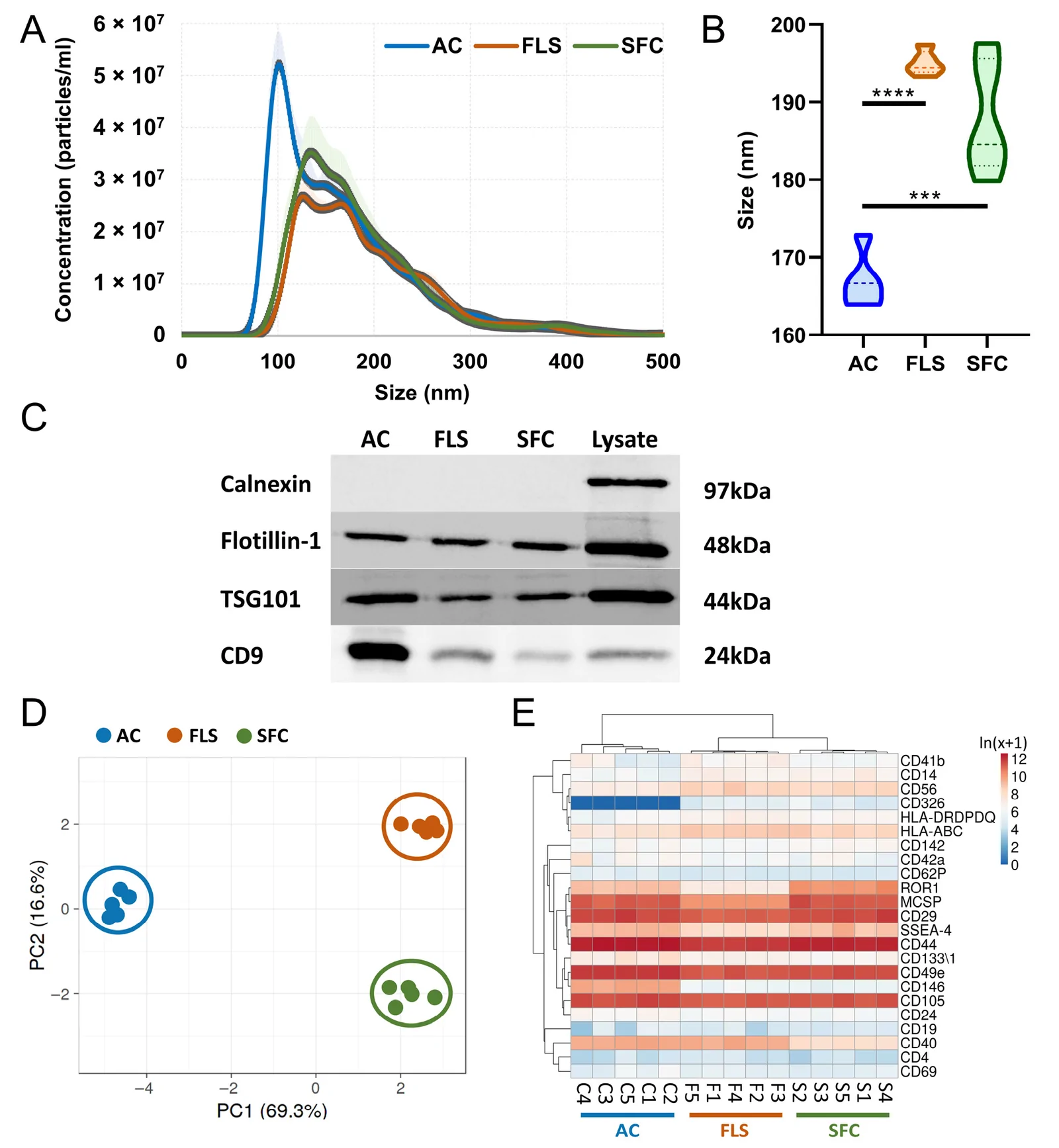
AC-, FLS- and SFC-released EVs phenotype characterization. (**A**) EVs averaged concentration and size. N = 5 for each EV type. Error bars indicate SD. (**B**) Particle size analysis using NTA. Size range results are displayed as violin plots. N = 5, *** for *p*-value ≤ 0.001 and **** ≤0.0001. (**C**) Western blot analysis of common EV markers (Flottilin-1, TSG101 and CD9) and cell organelle markers (Calnexin) in AC-, FLS- and SFC-EVs. Cell lysate is used as a control for positive signal. (**D**) Principal component analysis of the AC, FLS- and SFC-EVs based on their immunophenotype. The X and Y axes show PC1 and PC2, which explain 69.3% and 16.6% of the total variance, respectively. (**E**) Heat map of hierarchical clustering analysis of the ln(x + 1)-transformed fluorescence values of detected antigens in AC-, FLS- and SFC-EVs, with a sample clustering tree at the top. Absolute expression levels reflect the color scale: red shades: high expression levels; blue shades: low expression levels.

**Figure 3:**
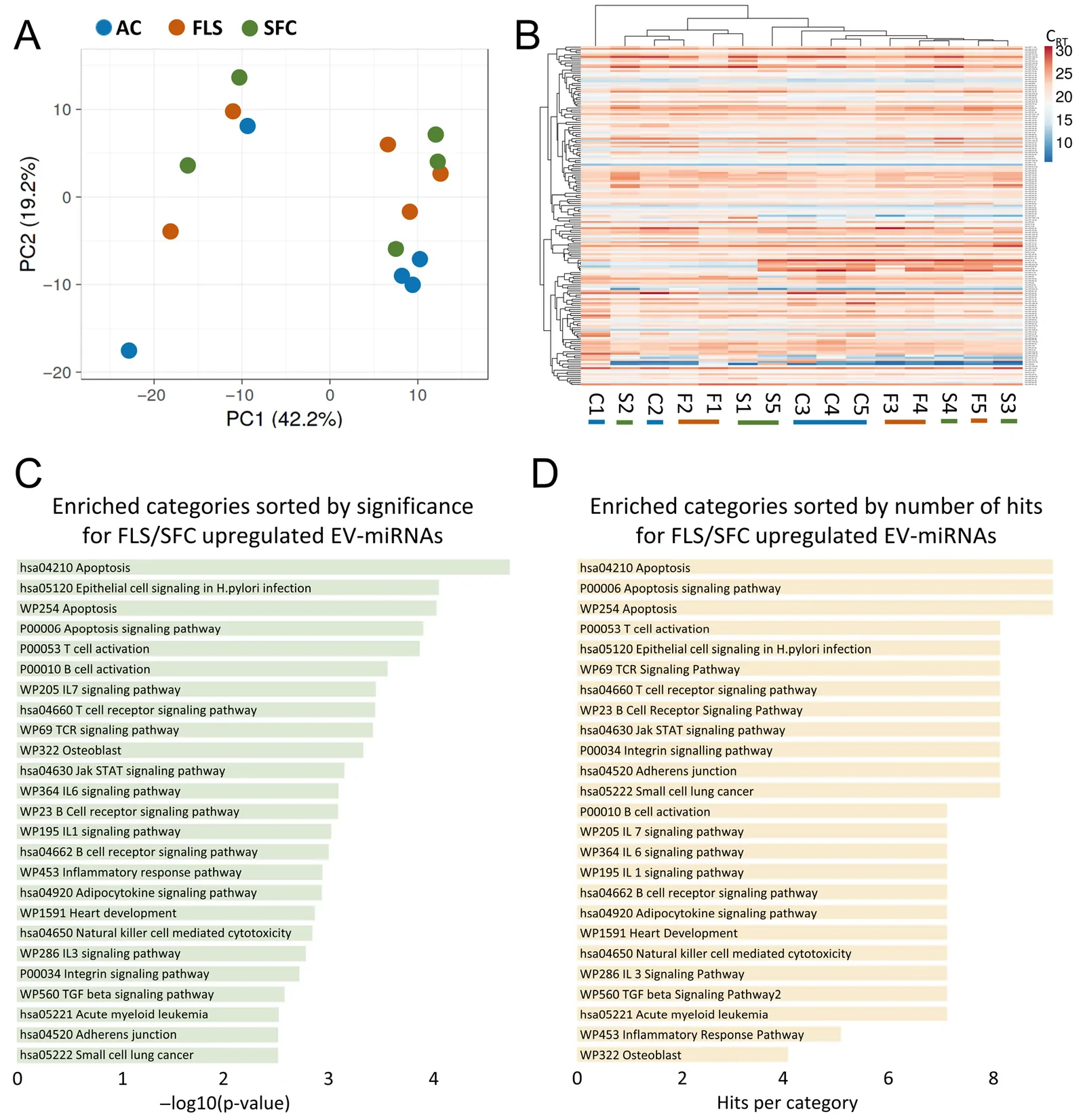
AC-, FLS- and SFC-released EV-miRNAs characterization. (**A**) Principal component analysis of the AC, FLS- and SFC-released EV-miRNAs based on their abundance as normalized C_RT_ values. The X and Y axes show PC1 and PC2, which explain 42.2% and 19.2% of the total variance, respectively. (**B**) Heat map of hierarchical clustering analysis of the EV-miRNAs normalized C_RT_ values in AC-, FLS- and SFC-EVs, with a sample clustering tree at the top. Absolute expression levels reflect the color scale: red shades: high expression levels; blue shades: low expression levels. (**C**) miRNA Enrichment and Annotation for FLS/SFC upregulated EV-miRNAs sorted by significance. hsa indicates KEGG pathway identifier; WP indicates WikiPathways database identifier. (**D**) miRNA Enrichment and Annotation for FLS/SFC upregulated EV-miRNAs sorted by number of hits. hsa indicates KEGG pathway identifier; WP indicates WikiPathways database identifier.

**Figure 4:**
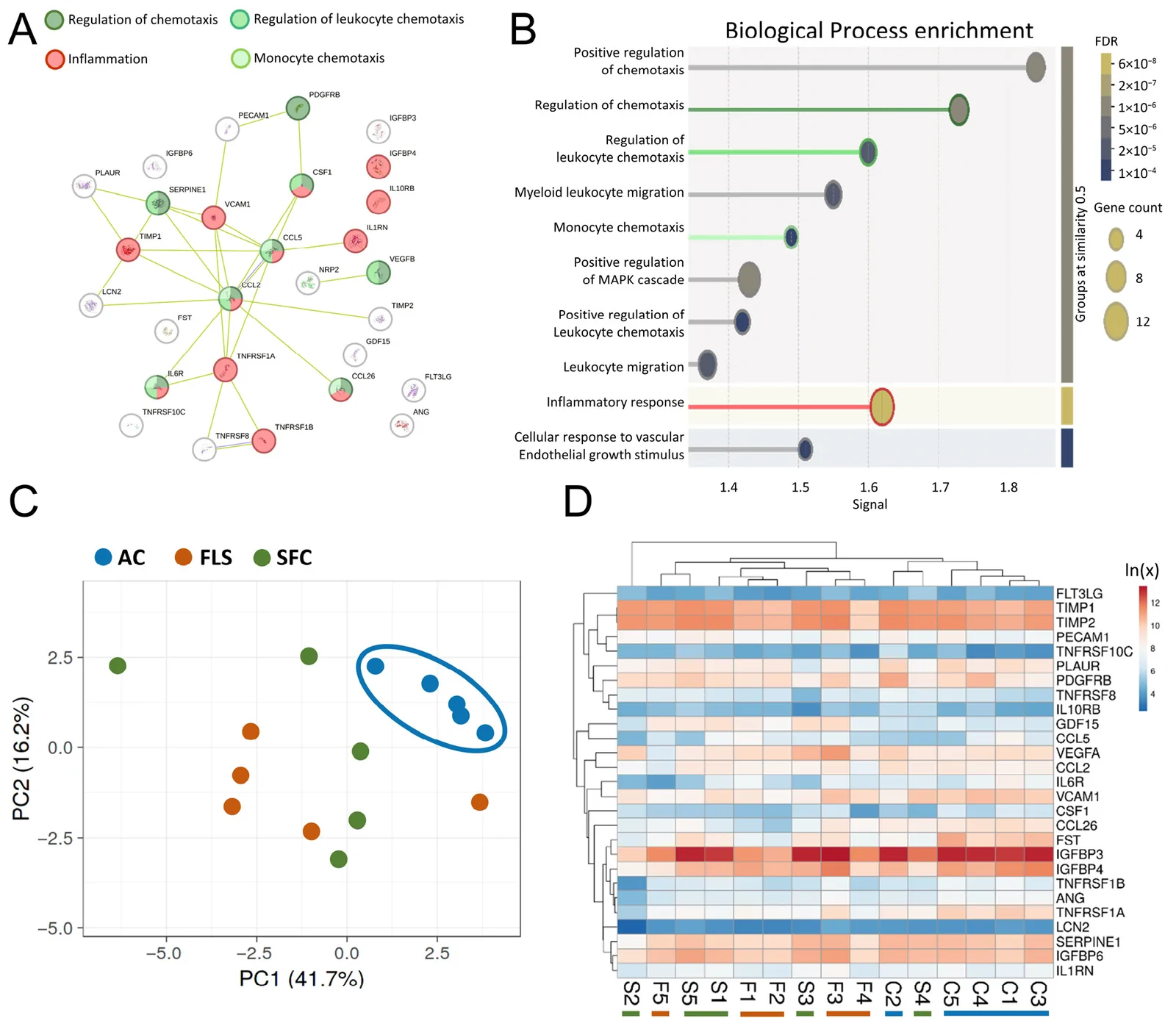
AC-, FLS- and SFC-released factors characterization. (**A**) Protein–protein interaction network for identified secreted factors mined using STRING, and related to inflammation and chemotaxis terms. Empty nodes: proteins of unknown 3D structure; filled nodes: known or predicted 3D structure. (**B**) Biological process enrichment for identified secreted factors mined using STRING. The top 10 GO terms are shown, with a color code: red and shades of green related to terms highlighted in panel (**A**). (**C**) Principal component analysis of the AC-, FLS- and SFC-released factors based on their abundance as ln(x)-transformed pg/mil values. The X and Y axes show PC1 and PC2, which explain 41.7% and 16.2% of the total variance, respectively. (**D**) Heat map of hierarchical clustering analysis of the released factors based on their abundance as ln(x)-transformed pg/mil values in ACs, FLSs and SFCs, with a sample clustering tree at the top. Absolute expression levels reflect the color scale: red shades: high expression levels; blue shades: low expression levels.

**Figure 5:**
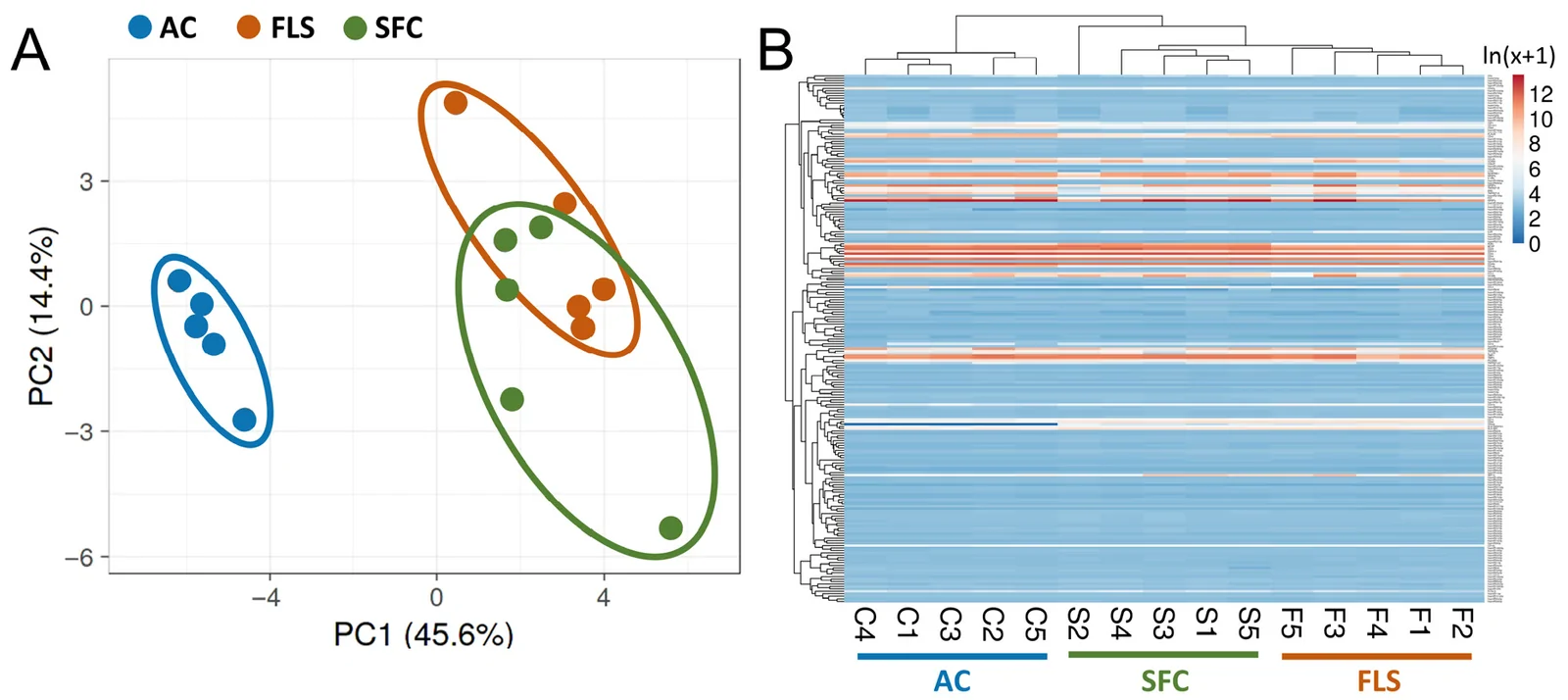
AC-, FLS- and SFC-released secretomes overall characterization. (**A**) Principal component analysis of the AC-, FLS- and SFC-released secretomes (EVs immunophenotype, EV-miRNAs and soluble factors) based on their abundance as ln(x + 1)-transformed values. The X and Y axes show PC1 and PC2, which explain 45.6% and 14.4% of the total variance, respectively. (**B**) Heat map of hierarchical clustering analysis of the AC-, FLS- and SFC-released secretomes (EV immunophenotype, EV-miRNAs and soluble factors) based on their abundance as ln(x + 1)-transformed values in ACs, FLSs and SFCs, with a sample clustering tree at the top. Absolute expression levels reflect the color scale: red shades: high expression levels; blue shades: low expression levels.

**Table 1:** Differentially detected surface markers in AC-, FLS- and SFC-EVs.

Factor	FLS vs. AC		SFC vs. AC		SFC vs. FLS		Function
CD326	+ ^1^	*	+ ^1^	*	0.86		Cell adhesion molecule
CD56	3.89	***	3.19	**	0.84		Cell adhesion molecule
HLA-ABC	3.11	****	2.40	***	0.81		Receptor
HLA-DRPDQ	2.77	**	1.87		0.77		Receptor
CD14	2.67	**	2.91	**	1.03		Receptor
CD40	0.94		0.20	****	0.23	****	Receptor
CD133\1	0.55		0.39	*	0.74		Glycoprotein
MCSP	0.32	****	1.11		3.57	****	Proteoglycan
SSEA-4	0.31	*	0.84		2.64		Glycosphingolipid
ROR1	0.16		3.31		21.32	**	Receptor tyrosine kinase
CD146	0.02	****	0.02	****	0.83		Cell adhesion molecule

^1^ + not present in AC-EVs. * for *p*-value ≤ 0.05, ** ≤0.01, *** ≤0.001 and **** ≤0.0001 coupled with ratio ≥ 2 or ≤0.5. FLS (fibroblast-like synoviocyte); SF (synovial fluid cell); AC (articular chondrocyte); CD (Cluster of Differentiation); HLA (Human Leukocyte Antigen − A/B/C); HLA-DRPDQ (Human Leukocyte Antigen − D Related/D Phosphate/D Glutamine); MCSP (Melanoma-Associated Chondroitin Sulfate Proteoglycan); SSEA (Stage-Specific Embryonic Antigen); ROR (Receptor Tyrosine Kinase Like Orphan Receptor).

**Table 2:** Ranking and functional relevance of the top 10 most abundant miRNAs in EVs.

miRNA	Ranking				Condition		Gene Ontology			
	MEAN	AC	FLS	SFC	OA	Inflammation	GO0022617	GO0050870	GO0043032	GO0006917
hsa-miR-636	1	1	2	2						
hsa-miR-551b-3p	2	9	1	1						
hsa-miR-24-3p	3	2	3	4			X	X	X	X
hsa-miR-520e-3p	4	3	6	3						
hsa-miR-125b-5p	5	6	4	5	X	X	X	X	X	X
hsa-miR-21-5p	6	4	5	6	X	X	X	X	X	X
hsa-miR-100-5p	7	5	8	8	X	X	X	X		X
hsa-miR-193b-3p	8	8	7	7			X			X
hsa-miR-99a-5p	9	7	9	9						X
hsa-miR-221-3p	10	10	11	10	X		X			X
*p*-value					7.57 × 10^−5^	8.13 × 10^−4^	0.00398	6.21 × 10^−4^	0.0124579	0.0126197

MEAN is the ranking in order of detection of the whole dataset without cell-source division. GO0022617 ECM disassembly; GO0050870 positive regulation of T cell activation; GO0043032 positive regulation of macrophage activation; GO0006917 induction of apoptosis.

**Table 3:** Differentially abundant miRNAs in EVs.

miRNA	FLS vs. AC		SFC vs. AC		SFC vs. FLS		miRNA	FLS vs. AC		SFC vs. AC		SFC vs. FLS	
hsa-miR-199a-3p	52.49		3.78	*	1.43		hsa-miR-146b-5p	0.96		0.25	*	0.90	
hsa-miR-181a-5p	17.38	**	26.05	**	1.57		hsa-miR-99a-3p	0.82		0.32	*	0.46	
hsa-miR-340-5p	6.08	*	7.01	*	1.26		hsa-miR-155-5p	0.64		0.37	*	0.95	
hsa-miR-218-5p	4.82	*	4.34	*	0.89		hsa-miR-128-3p	0.62		0.38	*	0.79	
hsa-miR-34c-5p	4.60	**	8.46	***	1.67		hsa-miR-140-3p	0.55		0.40	*	0.88	
hsa-miR-491-5p	3.76	**	2.70	*	0.70		hsa-miR-224-5p	0.55		0.30	***	0.59	
hsa-miR-29b-3p	3.68	*	5.36	**	1.94		hsa-miR-455-5p	0.51		0.18	**	0.48	
hsa-miR-199a-5p	3.03	*	2.85	*	1.10		hsa-miR-539-5p	0.51		0.34	**	0.73	
hsa-miR-34a-5p	2.87	**	4.05	**	1.36		hsa-miR-125b-1-3p	0.50	*	0.79		1.68	
hsa-miR-615-3p	2.52		7.47	*	3.22		hsa-miR-487b-3p	0.49	**	0.47	**	1.02	
hsa-miR-374a-5p	2.47		3.75	*	1.85		hsa-miR-323a-3p	0.48	***	0.51		1.10	
hsa-miR-29c-3p	2.21	**	2.38	***	1.12		hsa-miR-410-3p	0.46	*	0.48	*	1.13	
hsa-let-7d-5p	2.12		2.41	*	1.28		hsa-miR-134-5p	0.45	**	0.52		1.20	
hsa-miR-29a-3p	1.92		2.55	**	1.34		hsa-miR-337-5p	0.42	*	0.26	**	0.78	
hsa-miR-374b-5p	1.84		2.69	**	1.54		hsa-miR-543	0.41	**	0.32	**	0.96	
hsa-miR-101-3p	1.79		3.18	*	2.06		hsa-miR-495-3p	0.41	*	0.39		1.24	
hsa-miR-199b-5p	1.71		3.81	***	2.22		hsa-miR-335-5p	0.40	*	0.38	*	0.94	
hsa-miR-758-3p	1.70		4.14	*	3.43		hsa-miR-342-3p	0.39	**	0.31	***	0.83	
hsa-miR-26a-5p	1.65		2.53	***	1.65		hsa-miR-376c-3p	0.38	****	0.49	***	1.31	
hsa-miR-34a-3p	1.42		2.33	*	1.75		hsa-miR-140-5p	0.31	****	0.50	***	1.68	
hsa-miR-28-5p	1.38		2.73	*	2.05								
hsa-miR-10b-5p	1.19		3.22	**	2.82	*							

* for *p*-value ≤ 0.05, ** ≤0.01, *** ≤0.001 and **** ≤0.0001, coupled with ratio ≥ 2 or ≤0.5.

**Table 4:** Ranking and functional relevance of the top 10 most abundant soluble factors.

Factor	Ranking				Function	Role in OA
	Mean	AC	FLS	SFC		
IGFBP3	1	1	1	1	Secreted growth factor-binding glycoprotein	Pro-OA (Catabolism)
TIMP2	2	2	2	2	Secreted metalloproteinase inhibitor	Anti-OA (ECM protection)
TIMP1	3	4	3	3	Secreted metalloproteinase inhibitor	Anti-OA (ECM protection)
IGFBP4	4	3	4	5	Secreted growth factor-binding protein	Pro-OA (Catabolism)
IGFBP6	5	5	5	4	Secreted growth factor-binding protein	Pro-OA (Inflammation)
SERPINE1	6	6	7	6	Secreted serine protease inhibitor	Pro-OA (Synovial fibrosis)
VEGFA	7	12	6	7	Secreted dimeric angiogenic growth factor	Pro-OA (Angiogenesis)
PDGFRB	8	8	8	8	Transmembrane receptor tyrosine kinase	Pro-OA (Bone remodeling)
VCAM1	9	9	9	9	Transmembrane cell adhesion glycoprotein	Pro-OA (Inflammation)
FST	10	7	13	10	Secreted activin-binding glycoprotein	Anti-OA (Chondroprotection)

MEAN is the ranking in order of detection of the whole dataset without cell-source division.

**Table 5:** Ranking and functional relevance of differentially abundant soluble factors.

Factor	FLS vs. AC		SFC vs. AC		SFC vs. FLS		Function	Role in OA
IGFBP4	0.62		0.35	*	1.25		Secreted growth factor-binding protein	Pro-OA (Catabolism)
PLAUR	0.42	*	0.36	**	1.00		GPI-anchored cell surface receptor	Pro-OA (ECM degradation)
IL6R	0.40		0.31	*	1.27		Transmembrane/soluble cytokine receptor	Pro-OA (Inflammation)
TNFRSF1A	0.37		0.17	*	0.92		Transmembrane death domain receptor	Pro-OA (Inflammation)
CCL26	0.30	**	0.28	**	2.46		Secreted chemotactic cytokine	Pro-OA (Immune recruitment)
FST	0.16	*	0.24		2.42		Secreted activin-binding glycoprotein	Anti-OA (Chondroprotection)

* for *p*-value ≤ 0.05 and ** ≤0.01, coupled with ratio ≥ 2 or ≤0.5.

## Data Availability

The original data presented in the study are openly available in the Center for Open Science, Inc., at https://osf.io/27ac6/overview?view_only=a3868b8c9b8846f68ee08baed167b84d.

## Supplementary Materials

The following supporting information can be downloaded at: https://www.mdpi.com/article/10.3390/jcm15218403/s1, Table S1: Detected antigens in tested EVs; Table S2: Detected miRNAs in EVs; Table S3: miEAA–miRNA enrichment and annotation for FLS/SFC upregulated EV-miRNAs; Table S4: Detected soluble factors; Table S5: Enriched biological processes associated with soluble factors. Figure S1: AC samples’ responsiveness to Alcian Blue staining; Figure S2: TEM images of AC-, FLS- and SFC-EVs.

## Abbreviations

The following abbreviations are used in this manuscript:

OA	Osteoarthritis
AC	Articular Chondrocyte
FLS	Fibroblast-Like Synoviocyte
SFC	Synovial Fluid Cell
ECM	Extracellular Matrix
EV	Extracellular Vesicle
miRNA	microRNA
CD	Cluster of Differentiation
GO	Gene Ontology
NTA	Nanoparticle Tracking Analysis
PCA	Principal Component Analysis
